# Synthesis and Characterization of Reproducible Linseed Oil-Loaded Silica Nanoparticles with Potential Use as Oxygen Scavengers in Active Packaging

**DOI:** 10.3390/nano12183257

**Published:** 2022-09-19

**Authors:** Juan Felipe Alvarado, Daniel Fernando Rozo, Luis Miguel Chaparro, Jorge Alberto Medina, Felipe Salcedo-Galán

**Affiliations:** 1 Materials and Manufacturing Research Group (CIPP-CIPEM), Department of Chemical Engineering, Universidad de los Andes, Bogota 111711, Colombia; 2 Materials and Manufacturing Research Group (CIPP-CIPEM), Department of Mechanical Engineering, Universidad de los Andes, Bogota 111711, Colombia

**Keywords:** silica nanoparticles, oxygen scavenger, reproducibility, moisture independent

## Abstract

Commercially available oxygen scavengers used to prevent lipid autoxidation, microbial growth and enzymatic browning in food products present several issues, which include the usage of metals and their moisture dependence to work properly. We present the synthesis and characterization of a moisture-independent oil-based oxygen scavenging system comprised of linseed oil and silica nanoparticles. The system was synthesized via sol-gel chemistry and was characterized using morphological analysis (SEM, AFM, TEM, and N_2_ adsorption/desorption), oil-loading analysis (TGA), and surface analysis (ζ-potential and ATR-FTIR). Performance of the system was evaluated through headspace measurements and reproducibility of synthetic procedure was verified using six replicates. Nanoparticles showed the desired spherical shape with a diameter of (122.7 ± 42.7 nm) and mesoporosity (pore diameter = 3.66 ± 0.08 nm), with an encapsulation efficiency of 33.9 ± 1.5% and a highly negative ζ-potential (−56.1 ± 1.2 mV) in basic solution. Performance of the system showed a promising high value for oxygen absorption of 25.8 ± 4.5 mL O_2_/g of encapsulated oil (8.3 ± 1.5 mL O_2_/g of nanocapsules) through a moisture independent mechanism, which suggests that the synthesized system can be used as an oxygen scavenger in dry atmosphere conditions.

## 1. Introduction

Interaction between oxygen and various chemical compounds present in food products has been a major challenge of scientists looking to extend shelf-life and quality of products, as oxygen-mediated reactions play a key role in food deterioration [1]. On one side, reaction of oxygen with polyunsaturated fatty acids generates hydroperoxides that cause rancidity and have been associated with health dangers when ingested [1,2]. On the other, O_2_ triggers microbial growth and enzymatic browning causing further loss of shelf-life of the food product [3].

Oxygen scavengers (OS) are substances that absorb oxygen chemically or enzymatically and have been used in packaging systems to protect food against deterioration [4]. These types of systems in which a component is intentionally added to interact favorably with the food product through the release or absorption of an active component from or to the food product or environment [5,6] are called active packaging systems. 

The most common commercial oxygen scavengers are iron-based, which nowadays are implemented for mainly humid atmospheres due to the dependence of water in iron oxidation. However, special care has to be taken to avoid leakage of the active compound into the food product, which may not only alter the taste and smell, but also might impart a certain level of toxicity [7]. New technologies are, however, being studied every day which seek to minimize these possible adverse effects of iron-based scavengers such as self-activated [8] and non-metallic OS [3,9,10]. However, even some self-activated scavengers also rely on the presence of humidity on the sachet in which they are contained [7] or are pre-humidified before their use [11]. Other enzymatic scavengers also require the addition of water in order to perform optimally [7] and even modern activated carbon/ascorbic acid absorbers are reported to enhance their activity with water concentration [3]. Polyunsaturated fatty acids (PUFAs) have been reported to be a possible solution to metal toxicity and moisture related problems [7] due to the presence of carbon-carbon double bonds in their structure, which allow their oxidation via free-radicals through a water independent mechanism [12]. In this way, not only do PUFAs demonstrate efficient oxygen absorption kinetics [13,14], but also considerably less toxicity than metal based OS. 

Besides selecting or synthesizing the appropriate OS active component, other challenges also arise when deciding on how to incorporate the scavenger in the packaging system. In this process, special care must be taken to avoid a significant alteration of the properties of the package, and simultaneously, protect the integrity of the OS from the high temperature and stress present during typical processing methods implemented in the manufacturing of flexible or rigid plastic packages. One alternative to ensure this balance is to nanoencapsulate the OS inside a matrix that can be immobilized in the polymeric matrix of the package. Nanoencapsulation, besides protecting the OS to high shear and temperature operations, promotes an even distribution of the OS across the matrix and has proven to have little (if not a positive) effect on the mechanical properties of several polymers used today in food packaging [15,16]. This approach also makes it highly unlikely for the OS scavenger to migrate to the food product, preventing problems associated with toxicity or alteration of the quality of the product. 

Nanoencapsulation of oils containing high concentration of PUFAs (e.g., linseed oil) has been reported using a variety of encapsulating agents. For example, Miguel et al. [17] prepared linseed oil loaded microcapsules of poly (urea-formaldehyde). They were able to synthesize microcapsules of 1–20 µm using ultrasonication and poly (vinyl) alcohol and sodium dodecyl benzene as stabilizers. However, the system presented some disadvantages such as a high pH dependence on the mechanical behavior of the microcapsule, the need for two different stabilizers during the synthesis process, and the inability to prepare nanoscale capsules. In the scope of active packaging systems, major drawbacks can be encountered with this approach, which include the possible thermal and mechanical degradation of the microcapsules due to polymer processing techniques. Alternatively, Röcker et al. [18] implemented modified calcium carbonates as immobilizing agents for linoleic acid and oleic acid (two unsaturated fatty acids present in linseed oil). The porous internal structure consisting of hydroxyapatite and calcium carbonate endows the carrier of thermal and mechanical stabilization; however, scanning electron microscopy images show irregular carrier shapes and sizes in the micro range. This presents a major issue in the active packaging field as these irregular shapes are known to reduce mechanical properties of nanocomposites [19].

An attractive alternative for the nanoencapsulation of oils in active packaging system is the use of silica nanoparticles synthesized via sol-gel template-based chemistry due to its high versatility and simple approach [20]. Loaded silica nanoparticles synthesized using this method can be easily tailored and give a rigid support to the OS active molecule, which not only provides protection and an efficient distribution in the carriers, but also an easier manipulation of the OS and its incorporation in polymeric matrices. Silica also endows the OS system of mechanical, thermal, and chemical stability as it provides a rigid and strongly bound support for the OS [21,22,23]. However, one disadvantage of this method is the huge number of factors that play a key role during nanoparticle synthesis (precursor concentration, template concentration, shear-rate, stability, pH, catalyst concentration, aging time, among others) and during post synthesis methods (drying, purification, functionalization, among others). This requires having a careful control of the experiment in order to reproduce experimental results [24]. Therefore, we believe current research using sol-gel chemistry should present detailed reproducible protocols to ensure an accurate development of new science and technologies.

The aim of this work is to present a reproducible methodology for the synthesis of silica nanoparticles loaded with linseed oil and their detailed characterization and oxygen absorption performance analysis. Linseed oil was selected due to previous studies done by members of the research group which showed a high oxidation capacity [25]. The OS system was synthesized using sol-gel template-based chemistry. Results showed that spherical, mesoporous and highly tunable SiO_2_ nanoparticles loaded with linseed oil were successfully synthesized in a reproducible manner. The obtained OS system showed a promising moisture independent oxygen absorption value, which conveys their potential application in active packaging systems.

## 2. Materials and Methods

### 2.1. Reagents

Analytical grade ethanol (99.5% PanReac AppliChem, Barcelona, España), cetrimonium bromide (CTAB, 98% Sigma Aldrich, Darmstadt, Germany), tetraethoxysilane (TEOS, 98% Alfa Aesar, Heysham, United Kingdom), and ammonia solution (25% PanReac AppliChem) were implemented for nanoparticle synthesis. Linseed oil (LO, gas chromatography tested) was bought from Produquímica de Colombia S.A.S (Bogota, Colombia) and was used as the encapsulated active agent. Analytical grade chloroform (99% PanReac AppliChem) and prepared 2M NaOH (≥99%, pellets, Merck KGaA, Darmstadt, Germany) solution were implemented in post-synthesis procedures. All the above reagents were used as received.

### 2.2. Nanoparticle Synthesis 

Loaded silica nanoparticles (L-NPs) were synthesized using sol-gel emulsion templating chemistry [26,27]: 0.48 g (0.0013 mol) of CTAB were dissolved in a solution of 85.5 mL of deionized water and 36 mL of ethanol under constant magnetic stirring at 700 rpm until complete dissolution. After that, 1.5 mL of LO were added dropwise to the mixture and left for 15 min under the same stirring speed to form a pale white emulsion. Then, 6 mL (0.027 mol) of TEOS were slowly added to the flask and left for 5 min at constant stirring to allow migration of SiO_2_ precursor to surfactant micelle. Lastly, 3 mL of ammonia solution were slowly added, and the mixture was left at 700 rpm for 24 h at room temperature. The resulting L-NPs were centrifuged for 10 min at 4000 rpm and dried in a vacuum oven at 80 °C for 4 h. Dried L-NPs were grinded using a mortar and pestle to obtain a white fine powder. 2.34 g ± 0.05 g of white fine powder was produced per reaction. The previously described procedure arises from previous studies done by research group members [28].

Empty silica nanoparticles (E-NPs) were produced for N_2_ absorption/desorption and ζ-potential measurements to avoid possible interaction of LO and surfactant with the test. E-NPs were prepared from L-NPs by the means of four sequential processes. First, L-NPs were washed three times with analytical grade ethanol. Then, washed nanoparticles were resuspended in analytical grade ethanol and placed in a sonication bath at 40 °C and 28 kHz for 15 min to remove surfactant template [29]. A 24-h Soxhlet extraction with analytical grade ethanol was done afterwards, followed by a 24-h Soxhlet extraction with chloroform to ensure complete oil and surfactant removal. Lastly, E-NPs were calcinated at 550 °C for 6 h.

### 2.3. Nanoparticle Characterization 

The morphologies and sizes of dried nanoparticles were characterized using scanning electron microscopy (SEM, Lyra 3, TESCAN a.s.). Dried L-NPs were sputter coated with a thin layer of gold to increase sample conductivity Particle size distribution was obtained from SEM images using ImageJ. A total of 150 nanoparticles per sample were used for the particle size distributions. Qualitative characterization of nanoparticle surface was done using atomic force microscopy (AFM, MFP-3D-BIO, Asylum Research, Oxford Instruments, Abingdon, United Kingdom) and transmission electron microscopy (TEM, JEOL 1400 plus). For TEM, a small volume of an aqueous solution containing 5 mg/mL of nanoparticles was placed over the grid and allowed to dry at room temperature for two days. After that, the observation was performed. 

Oil loading and oil retention were determined by thermogravimetric analysis (TGA) on a simultaneous analyzer (SDT-Q600, TA Instruments, Dallas, TX, USA). In each test, the material was heated from room temperature to 700 °C at a heating rate of 10 °C min^−1^ under a UHP nitrogen flow (rate: 100 mL min^−1^). Oil loading was associated with the mass fraction of oil compared to the mass of nanocapsules (obtained directly from the TGA as the mass percent loss associated with oil decomposition), while oil retention was associated with the amount of oil in the powder compared to the amount of oil added initially to the reaction mixture. To calculate encapsulation efficiency, the L-NPs were washed with analytical grade ethanol three times and dried in a vacuum oven at 80 °C for 4 h. The weight difference between non-washed and washed L-NPs was measured and used to calculate encapsulation efficiency. This procedure was established by Jayanudin et al. [30]. Oil loading, oil retention, and encapsulation efficiency were calculated using Equations (1)–(3), where ${\;x}_{\mathrm{loss}\left(\mathrm{TGA}\right)}$ is the mass fraction associated with oil decomposition, $m_{\mathrm{LO}}$ is the mass of oil contained in the amount of dry powder obtained per reaction, $m_{L-\mathrm{NPs}}$ the mass of dry powder, $m_{T-\mathrm{LO}}$ the mass of LO added to the reaction flask, and $m_{E-\mathrm{LO}}$ the mass of encapsulated LO:

(1) $${\mathrm{Oil}\;\mathrm{loading}=x}_{\mathrm{loss}\left(\mathrm{TGA}\right)}\times 100$$

(2) $$\mathrm{Oil}\;\mathrm{retention}=\mathrm{Oil}\;\mathrm{loading}\;\times \left(\frac{m_{\mathrm{LO}}}{m_{\mathrm{T-LO}}}\right)$$

(3) $$\mathrm{Encapsulation}\;\mathrm{efficiency}=\left(\frac{m_{\mathrm{E-LO}}}{m_{\mathrm{T-LO}}}\right)\times 100$$

ζ-potential was measured using a 0.5% *w*/*v* suspension of E-NPs in deionized water in a Zetasizer Nano ZS from Malvern Instruments. pH was adjusted to a value of 10 with a 2M NaOH solution to promote surface silanol group deprotonation. ζ-potential measurements were complemented with Fourier Transformed Infrared Spectroscopy (FT-IR) using a Shimadzu IRTracer-100 spectrometer equipped with a PIKE Technologies Diamond Attenuated Total Reflectance (ATR) accessory. Nitrogen adsorption/desorption isotherms were recorded in a Quantachrome 1C Autosorb gas sorption analyzer. Samples were degassed at 300 °C for 3 h followed by nitrogen adsorption/desorption at −196 °C. Total surface area was calculated using multipoint BET analysis, whereas the NLDFT adsorption branch model for N_2_ in silica was used to calculate pore size distribution and pore volume, assuming a cylindrical pore shape. All the analysis were carried out using Quantachrome Autosorb 1C computer software for Windows. 

### 2.4. Performance Analysis

Oxygen absorption was quantified using a headspace oxygen analyzer model 901 from Quantek Instruments, Inc (Grafton, IL, USA). In addition, 1.2 g of dried L-NPs were packed in 8 cm × 18 cm triple layer bags (polypropylene, metallic polyester, and LDPE). The bags were vacuumed, and a known quantity of dry air (120 mL) was added to each bag. Headspace oxygen measurements were performed destructively to avoid gas leaks due to sequential punctures. Empty bags with UHP nitrogen instead were used as a leak control during the experiment. The experiment was carried out by triplicate.

### 2.5. Reproducibility Evaluation

Six replicate experiments were done to assess each characterization parameter in L-NPs (or E-NPs, given the case), except for high-cost experiments (porosimetry), in which only three replicate experiments were done. Relative standard deviation of less than 10% was established as the reproducibility criterion for each parameter, except for pore volume and BET superficial area, due to their high sensitivity. In the latter two parameters, a relative standard deviation of less than 15% was established as the reproducibility criterion. Nanoparticle size distribution reproducibility was assessed by means of a normal distribution fitting to the histograms for each sample. From the fitting, mean and standard deviation were obtained and compared between replicates. The criteria of relative standard deviation of less than 10% remained as a reproducibility criterion. 

## 3. Results and Discussion

### 3.1. Nanoparticle Characterization

**Figure 1:**
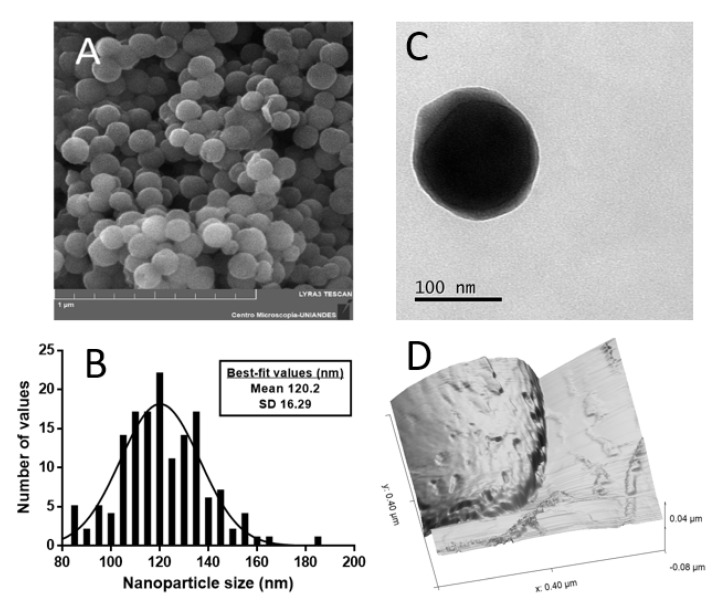
(**A**) SEM image of L-NPs synthesized via sol-gel template-based chemistry; (**B**) nanoparticle diameter histogram for a representative sample and the best fit values for a normal distribution; (**C**) TEM image of a single E-NPs showing the core-shell structure; (**D**) AFM image of E-NPs showing the presence of nanopores in the surface.

Morphology of nanoparticles is a key factor in active packaging applications. Cubic and hexagonal geometries, although they present higher surface area and hence higher oxygen diffusion, their interaction in polymeric nanocomposites might result in a higher level of aggregation due to their increased surface free energy [31]. Aggregation of nanofiller is a major inconvenience in nanocomposite synthesis since it causes a significant loss of bulk failure strength and bulk stiffness [32]. Spherical shape, on the contrary, presents higher stability and has been shown to reduce stress concentration in polymeric matrices leading to materials with enhanced mechanical properties [16,19,33,34]. Figure 1a displays the SEM images of L-NPs (SEM images of other replicates can be seen in Figure S1). The nanoparticles present a defined spherical shape, a grouped mean diameter of 122.7 ± 42.7 nm, and a low level of aggregation when compared to previously studied “two-pot” methods [35], which endow the obtained material of high potential on the active packaging sector. Figure 1b shows a representative histogram of a sample. Each sample showed a broad nanoparticle diameter distribution which might be related to a low temperature synthesis. This behavior becomes more evident when calculating the grouped standard deviation (42.7 nm). Monodispersity can be enhanced in future works by changing precursor concentration and increasing the temperature of synthesis [36]. Figure 1c shows the TEM micrograph of E-NPs, in which hollow nanoparticles can be observed. An irregular shell thickness was noticed for several E-NPs which might be due to precursor accumulation in a particular micelle location. This imaging verifies that the obtained system follows a template-based synthesis, which allows OS encapsulation. Figure 1d displays the AFM image of single E-NPs where the desired pores can be appreciated in the surface. These pores will serve as oxygen diffusing channels that enhance mass transfer from surrounding oxygen to the core of the nanoparticle. Nanoparticle and pore size can be roughly estimated from Figure 1d due to strong tip convolution effects.

**Figure 2:**
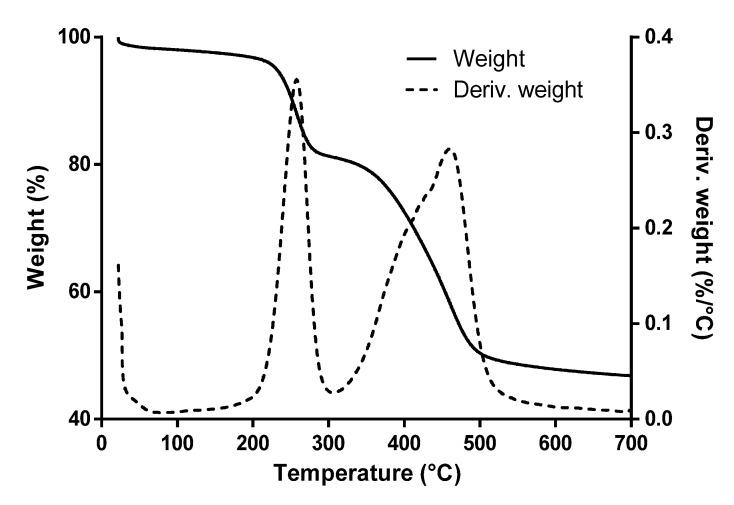
TGA curve for L-NPs showing thermal decomposition of CTAB (200–300 °C) and LO (300–550 °C). TGA was done under UHP nitrogen atmosphere.

Representative TGA of one L-NPs replicate is shown in Figure 2. Two prominent peaks can be seen in the weight derivative indicating important weight losses. The first is associated with decomposition of the surfactant CTAB and occurs in a temperature range of 200–300 °C (deriv. weight peak at 257 °C). CTAB is reported to decompose entirely over 300 °C [37]. The second is associated with the decomposition of LO, which takes place in a temperature range of 300−550 °C (deriv. weight peak at 460 °C). Correspondence of this weight loss was verified through pure LO TGA (see Figure S2) and with existent literature [17,38]. Oil encapsulation was verified using Figure S2. Here, a weight derivative peak associated with oil decomposition can be seen at 406 °C with a sharp peak, while in Figure 2 the peak is shifted to 460 °C and is broader. This phenomenon is associated with a thermal stabilization of the oil due to the presence of the silica shell around it. Thermal stabilization of oils due to encapsulation has been reported by several authors [39,40].

Oil loading was determined by quantification of weight percentage change in second loss for each replicate and was found to be 34.0 ± 2.3%. However, the oil loading does not reflect the amount of efficiently encapsulated oil, but rather the oil content in the solid sample of nanoparticles, which include free oil and encapsulated oil. To determine the oil uptake, two parameters were calculated: oil retention and encapsulation efficiency. The first parameter showed a value of 57.4 ± 3.2%, which indicated that more than half of the oil added to the reaction mixture was taken up in the nanoparticle powder. Encapsulation efficiency had a lower value (33.9 ± 1.5%) indicating that a certain amount of the oil taken up was free oil while only about one third of the oil added to the reaction mixture was efficiently encapsulated. Encapsulation efficiency value was compared to analogous encapsulation procedures using silica mesoporous material [41,42] and was found to present a borderline high value. Increased values of this parameter have been reported by using more complex experimental procedures [43]. 

**Figure 3:**
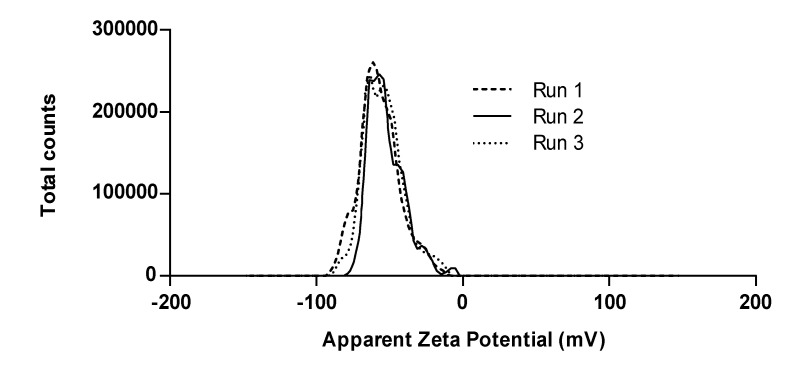
ζ-potential distribution of a 0.5%*w*/*v* solution at pH 10 for a representative L-NPs sample.

ζ-potential has been a widely used parameter to indirectly characterize surface polarity of nanoparticles [44,45]. This parameter is of special importance in most nanoparticle applications since it will predict their stability and dispersion in a given medium. In polymeric nanocomposites used for oxygen scavenging purposes, the interaction between polymer matrix and nanoparticle is key to promoting a high dispersion of nanofiller. Several authors have reported a significant loss in mechanical properties of nanocomposites due to nanoparticle agglomeration, as a consequence of unfavorable interaction between the surface of the nanoparticle and the matrix [33,46,47]. The interactions between matrix and nanofiller are governed by intermolecular interactions; thus, the surface groups of the nanoparticle must be compatible with the polarity of the polymeric chains surrounding them to ensure an homogeneous distribution. This highlights the importance of surface characterization in nanofillers. At high or low values of pH, having either a highly positive or a highly negative ζ-potential indicates a polar surface group that probably undergo ionization [45]. However, if at high or low pH the ζ-potential does not show high absolute values, it means that the surface of the nanoparticle contains groups that are not polar or ionizable [48]. Figure 3 shows the ζ-potential distribution for E-NPs suspended in basic aqueous medium. A narrow zeta potential distribution with a maximum value close to −50 mV was seen for a representative sample, which indicates the presence of ionizable groups in the surface corresponding to free Si-OH. At high pH values, this group undergoes deprotonation, causing a highly negative ζ-potential. These results indicate the presence of free silanol groups in the nanoparticle’s surface, which provides reactive groups for functionalization with different capping agents, and therefore, allows the implementation in a wide variety of polymers with different degrees of polarity used for packaging in the food industry [49,50,51,52]. Mean ζ-potential was −56.1 ± 1.2 mV, which agrees with values reported in literature for silica nanoparticles [44,45,53]. 

**Figure 4:**
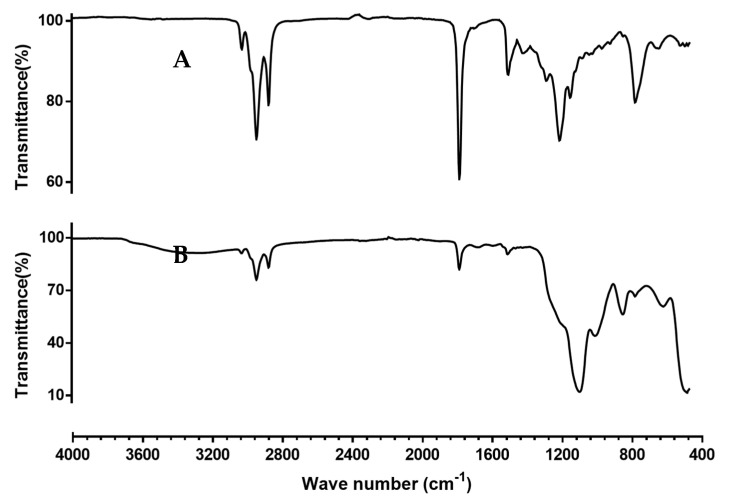
(**A**) ATR-FTIR spectrum of LO; (**B**) ATR-FTIR spectrum of a representative L-NPs sample.

Figure 4 shows the ATR-FTIR spectra for (a) linseed oil and (b) L-NPs. In Figure 4a, high intensity bands for aliphatic C-H bonds can be seen around 3000 cm^−1^. A carbonyl group from the fatty acids present in LO was also detected by a sharp peak in 1740 cm^−1^. In Figure 4b, asymmetric stretching bands of Si-O-Si and Si-OH bonds are visible at 1041 cm^−1^ and 952.84 cm^−1^ [54], respectively, indicating the presence of siloxane bond and the expected silanol group on the nanoparticle surface. A small and broad peak between 3600–300 cm^−1^ indicates the presence of a small fraction of water remaining after the drying process. Characteristic LO bands for C=O and aliphatic C-H bonds can be seen in L-NPs spectrum at 1743 cm^−1^ and 2800–3020 cm^−1^, respectively. These bands correspond to the linseed oil present on the nanoparticle sample. 

**Figure 5:**
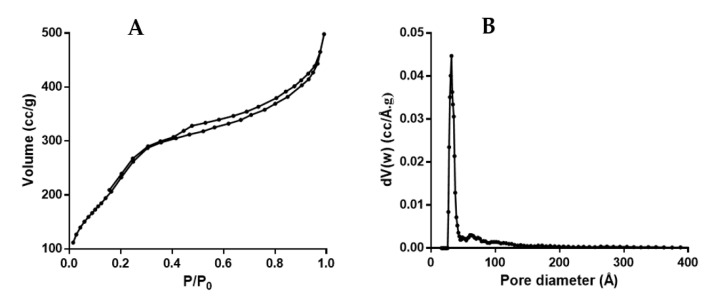
(**A**) Nitrogen adsorption/desorption isotherm at −196 °C for a representative E-NPs sample; (**B**) pore size distribution for a representative E-NPs sample using an NLDFT adsorption branch model for N2 in silica.

Figure 5a shows the nitrogen adsorption/desorption isotherms for a representative E-NPs sample. The shape and hysteresis of the isotherm indicate a type IV behavior, according to IUPAC characterization [55]. Average pore size was found to be 3.665 ± 0.08 nm, while total pore volume and BET superficial area values were 0.645 ± 0.08 cc/g and (7.5 ± 1.1) × 10^3^ m^2^/g, respectively. Figure 5b showed the abundance of pores exceeding 2.5 nm (mesopores), which agreed with the isotherm behavior. The presence of mesopores and micropores is preferred in the design of active packaging systems with oxygen scavenging properties due to increased flow of the gas across the cross-sectional area of diffusion. This enhanced mass transport was verified by Ferrero et al. [56] in analogous mesoporous materials for the reduction of oxygen.

### 3.2. Performance Analysis

**Figure 6:**
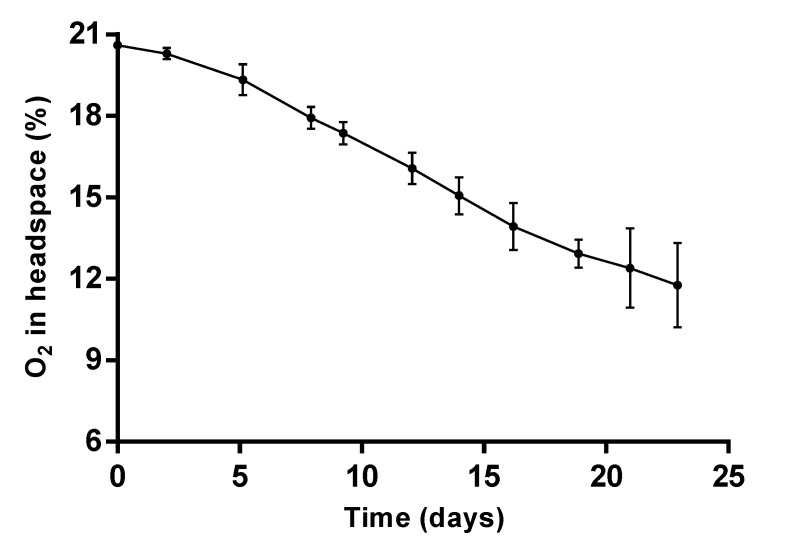
Oxygen in headspace analysis for the L-NPs system.

The oxygen absorption curve can be seen in Figure 6. A reduction of 8.9 percentual units was evidenced by day ~30 (550 h), indicating a reduction of more than 40% of the initial amount of oxygen present in a 120 mL sealed sample of air. Up to that moment, an absorption of 8.3 ± 1.5 mL O_2_/g of L-NPs was observed (25.8 ± 4.5 mL O_2_/g of encapsulated LO). Maximum oxygen absorption capacity (total amount of oxygen to be absorbed at infinite time) is yet to be determined; however, oxygen absorption up to 30 days can be used to compare our system to reported ones: commercial iron-based oxygen scavengers present more efficient absorption in the presence of moisture [57]; however, no oxygen absorption is reported for this type of scavenger in the absence of water. Other non-ferrous oxygen scavengers such as the mixture of activated carbon and sodium ascorbate reported by Lee et al. [3] show partial moisture independence, that is, they can absorb oxygen in dry atmospheres but at very low efficiencies (up to 3.33 mL of O_2_/g of non-encapsulated absorber). Other low moisture O_2_ absorbers like the reported by Gaikwad and Lee [9] based on pyrogallol are able to achieve up to 10.98 mL of O_2_/g of non-encapsulated absorber at 23 °C and 11% relative humidity, yet the problem regarding the water dependence is still present. To sum up, despite the long absorption time, the synthesized L-NPs possess a considerable moisture-free oxygen absorption, which make them a good candidate for active packaging systems in dry atmospheres. 

### 3.3. Reproducibillity

Reproducibility of the synthetic procedure was assessed by analyzing the characterization parameters of each one of the replicates. Table 1 shows a summary of the obtained result with a simple statistical analysis (individual values for each one of the replicates can be found in Table S1). The coefficient of variation (CV) is a statistical parameter used to compare the value of standard deviation with respect to the mean value. It is often used as a control for experiments to ensure low variability (i.e., higher reproducibility), where high values of CV will indicate high variation of data and low values, the opposite [58]. In this work, we determined the CV for each characterization parameter and established a limiting value to ensure reproducibility as mentioned in Section 2.5. As it can be seen, all the characterization parameters fulfill the reproducibility criteria, which indicate that the proposed synthesis and encapsulation protocol produce comparable results in each try.

## 4. Conclusions

The food industry faces a huge challenge related to oxidation of food in the presence of oxygen. Oxygen scavenging systems that can be adjusted to the several conditions in which a food can be packed should be designed in order to preserve all types of food. In the present work, we were able to design a moisture-independent oxygen scavenging system comprised of linseed oil and mesoporous silica nanoparticles, which can be used in active packaging systems. The system possesses high versatility (i.e., easy surface modification and practical incorporation in polymeric matrices), high porosity (enhanced oxygen diffusion), high oxygen absorption when compared to commercial systems in dry conditions, and it was demonstrated that they can be produced in a reproducible manner through a straightforward protocol based on sol-gel template-based chemistry. Further work should be aimed at increasing encapsulation efficiency and incorporating the nanoparticles in polymeric matrices that can prototype commercial active packaging. 

## Figures and Tables

**Table 1:** Reproducibility analysis for synthesis of L-NPs (or E-NPs, given the case).

Parameter	Mean	Standard Deviation	CV ^α^	Criteria Fulfilled
Mean_(ND)_ (size) (nm) ^β^	122.67	2.69	0.03	Yes
SD_(ND)_ (size) (nm) ^β^	17.27	0.74	0.04	Yes
Oil loading (%)	34.03	2.34	0.07	Yes
Oil retention (%)	57.35	3.23	0.06	Yes
Encapsulation efficiency (%)	33.92	1.46	0.04	Yes
ζ-potential (mV)	−56.05	1.19	0.02	Yes
Mean pore diam. (nm)	3.66	0.08	0.02	Yes
Mean pore vol. (cc/g)	0.64	0.08	0.12	Yes
BET Area (m^2^/g)	747.23	104.77	0.14	Yes

^α^ Dimensionless, ^β^ Mean and standard deviation of normal distribution fitting to size histograms.

## Data Availability

Not applicable.

## Supplementary Materials

The following supporting information can be downloaded at: www.mdpi.com/article/10.3390/nano12183257/s1, Figure S1: SEM image of L-NPs synthesized via sol-gel template-based chemistry; Figure S2: TGA curve for free LO showing thermal decomposition from 300 °C–500 °C. TGA was done under UHP nitrogen atmosphere; Table S1: Characterization parameters of L-NPs (or E-NPs, given the case) for each sample.
