# Supplementary material for: Synthesis and Characterization of Reproducible Linseed Oil-Loaded Silica Nanoparticles with Potential Use as Oxygen Scavengers in Active Packaging
Source: Nanomaterials (Basel). 2022 Sep 19;12(18):3257. doi: 10.3390/nano12183257 (PMC9502869; doi:10.3390/nano12183257)
Supplement: Supplementary file 1 [file nanomaterials-12-03257-s001.zip › nanomaterials-1869153-supplementary.pdf]

Supplementary Material

Synthesis and Characterization of Reproducible Linseed Oil-Loaded Silica Nanoparticles with Potential Use as Oxygen Scavengers in Active Packaging

Juan Felipe Alvarado [1,*], Daniel Fernando Rozo [1], Luis Miguel Chaparro [1], Jorge Alberto Medina [2], and Felipe Salcedo-Galán [1,*]

1 Materials and Manufacturing Research Group (CIPP-CIPEM), Department of Chemical Engineering,
Universidad de los Andes, 111711 Bogota, Colombia
2 Materials and Manufacturing Research Group (CIPP-CIPEM), Department of Mechanical Engineering,
Universidad de los Andes, 111711 Bogota, Colombia
* Correspondence: jf.alvarado10@uniandes.edu.co (J.F.A.); fesalced@uniandes.edu.co (F.S.G.);
Tel. (F.S.G.): +57-1-339-4949 (ext. 3942)

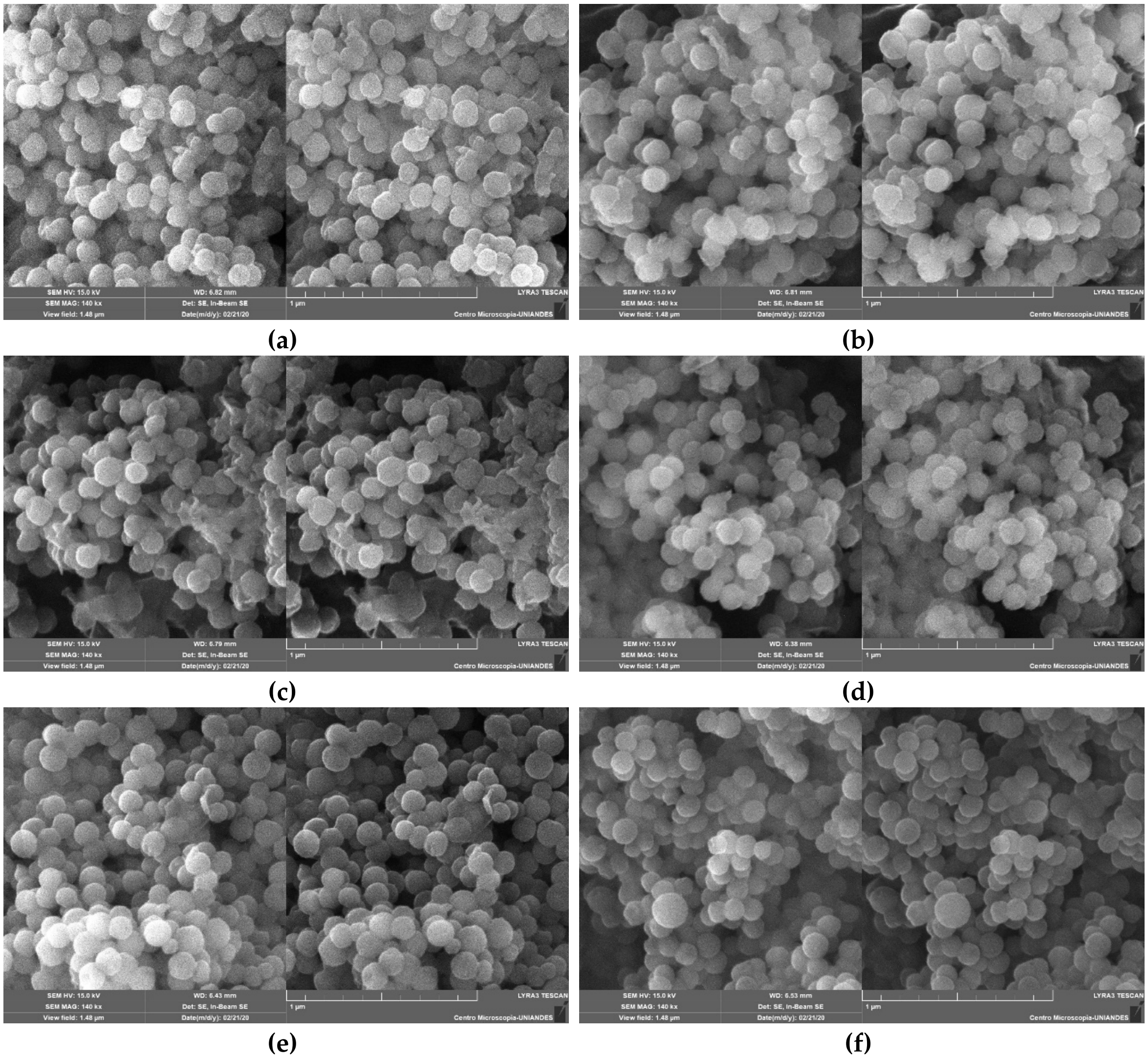

Figure S1. (a–f) SEM image of L-NPs synthesized via sol-gel template-based chemistry. Each set of two images correspond to a replicate of the experiment. Left side image corresponds to secondary electron (SE) detector, while right side image corresponds to in-beam secondary electron (In-Beam SE) detector. The measuring scale corresponds to 1μm.

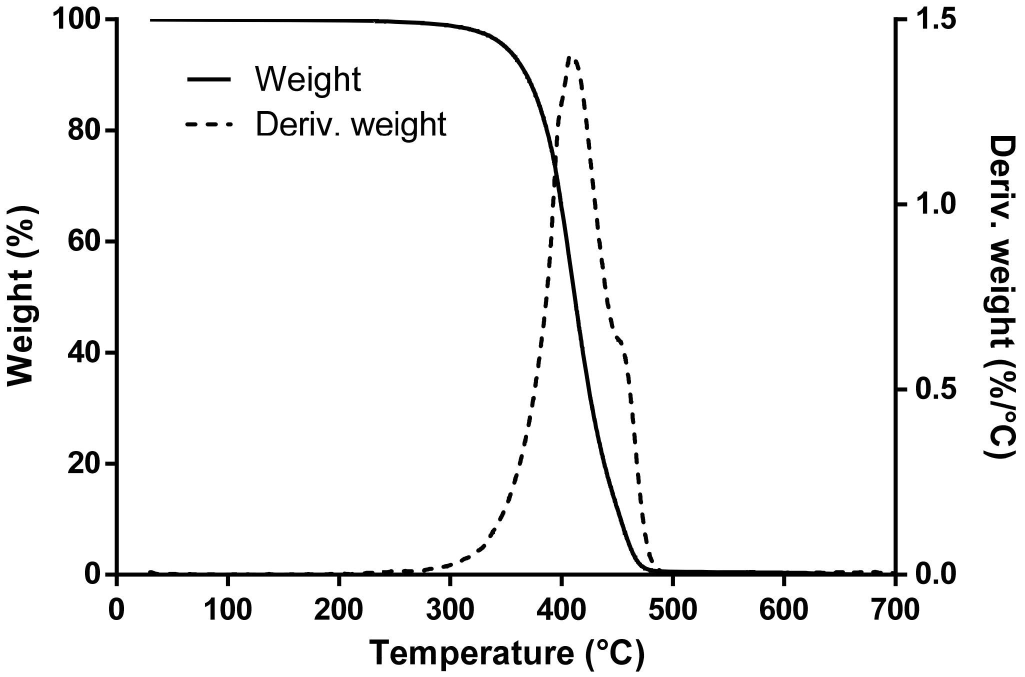

Figure S2. TGA curve for free LO showing thermal decomposition from 300°C-500°C. TGA was done under UHP nitrogen atmosphere.

Table S1. Characterization parameters of L-NPs (or E-NPs, given the case) for each sample. [1]CV stands for coefficient of variation and is expressed as a dimensionless quantity. The parameters $Mean_{(ND)}$ and $SD_{(ND)}$ correspond to the mean and standard deviation of each one of the normal distribution fittings done to each sample's histogram.

Sample	$Mean_{(ND)}$ (size) (nm)	$SD_{(ND)}$ (size) (nm)	Oil loading (%)	Oil retention (%)	Encapsulation efficiency (%)	ζ-potential (mV)	Pore diameter (nm)	Pore volume (cc/g)	BET Area (m^2/g)
**1**	119.15	18.55	35.29	58.41	33.67	−57.77	3.571	0.65	773.60
**2**	122.19	17.21	32.57	55.57	33.56	−55.20	3.713	0.56	631.80
**3**	124.87	16.99	31.03	54.12	32.97	−55.57	3.696	0.72	836.30
**4**	123.12	16.88	32.34	54.04	32.92	−56.97	-	-	-
**5**	120.39	16.40	36.62	61.16	36.46	−56.23	-	-	-
**6**	126.31	17.57	36.32	60.82	35.65	−54.53	-	-	-
$\bar{X}$	122.67	17.27	34.03	57.35	33.92	−56.05	3.66	0.64	747.23
**σ**	2.69	0.74	2.34	3.23	1.46	1.19	0.08	0.08	104.77
**CV[1]**	0.02	0.04	0.07	0.06	0.04	0.02	0.02	0.12	0.14
